# Crowd-Funding: A New Resource Cooperation Mode for Mobile Cloud Computing

**DOI:** 10.1371/journal.pone.0167657

**Published:** 2016-12-28

**Authors:** Nan Zhang, Xiaolong Yang, Min Zhang, Yan Sun

**Affiliations:** School of Computer and Communication Engineering, University of Science and Technology Beijing, Beijing, China; West Virginia University, UNITED STATES

## Abstract

Mobile cloud computing, which integrates the cloud computing techniques into the mobile environment, is regarded as one of the enabler technologies for 5G mobile wireless networks. There are many sporadic spare resources distributed within various devices in the networks, which can be used to support mobile cloud applications. However, these devices, with only a few spare resources, cannot support some resource-intensive mobile applications alone. If some of them cooperate with each other and share their resources, then they can support many applications. In this paper, we propose a resource cooperative provision mode referred to as "Crowd-funding", which is designed to aggregate the distributed devices together as the resource provider of mobile applications. Moreover, to facilitate high-efficiency resource management via dynamic resource allocation, different resource providers should be selected to form a stable resource coalition for different requirements. Thus, considering different requirements, we propose two different resource aggregation models for coalition formation. Finally, we may allocate the revenues based on their attributions according to the concept of the "Shapley value" to enable a more impartial revenue share among the cooperators. It is shown that a dynamic and flexible resource-management method can be developed based on the proposed Crowd-funding model, relying on the spare resources in the network.

## 1. Introduction

With the rapid development of mobile communication techniques, both the number of mobile terminals and the number of mobile applications are undergoing an exponential growth. Among the existing applications, some of them require high computational complexity.

Because both computational resources and battery power are limited for mobile terminals, the problem of resource shortages is unavoidable while running some computationally demanding applications [1]. To address the above-mentioned challenges, mobile cloud computing technology came into being by integrating the cloud computing techniques into the wireless mobile network [2,3], which is capable of offloading the burdens of both data storage and computing resources from mobile devices [4–6]. Mobile cloud computing can conserve the energy of mobile devices and reduce the execution time of mobile applications [7, 8].

In the network, the distributed spare resources can also be used to support mobile applications, which would be more efficient and less susceptible to network limitations than offloading data to remote data centers [9]. In a network, there are various devices carrying many spare resources, which can support different types of applications. Considering the insufficiency of resources in a single device, we propose a new resource cooperation mode referred to as "Crowd-funding". In the proposed "Crowd-funding" mode, devices with spare resources distributed in a network can be integrated to form cooperation coalitions, and then providing resources for mobile cloud services.

Moreover, the resource-management problem is also regarded as an important issue with regard to implementing the mobile cloud computing technique. To consider the particularity of different requests, we should enforce high flexibility and reliability on the resource management functionality. Moreover, a flexible resource-management scheme, as well as an extensible resource supply, is also required to accommodate the rapid development of mobile cloud computing techniques. To enforce high flexibility on the resource management functionality, we present two resource aggregation models according to the otherness of application requirements. Moreover, a fair revenue allocation method is proposed to share the revenue among the resource providers in the coalition [10]. The main contributions of this paper are as follows:

- A new resource cooperation mode named “Crowd-funding” is proposed. In Crowd-funding, geography-distributed devices with spare resources are able to cooperate with each other and aggregate into a resource coalition dynamically according to different application requirements. In return, they can receive some revenues according to their contributions.
- Two resource-aggregating models are presented, i.e., “Max-CR” and “Max-CQ”. The aggregating objective of “Max-CR” is to find out the resource coalition with the highest average revenue of unit resource, which is suitable for the requests without any specific service-level requirement. For the requests with some QoS (Quality of Service) requirements, the process of resource aggregation should be based on the “Max-CQ” model, whose aggregating objective is to find out the resource coalition with the highest QoS level.
- The revenue allocation among the resource providers should be implemented according to their contributions. Thus, the concept of the “Shapley value” is employed to enable the impartial revenue sharing among different providers.

The rest of this paper is organized as follows: Section 2 introduces the related works. Section 3 describes the Crowd-funding system. The models for resource aggregation and revenue allocation are proposed in Section 4 and Section 5, respectively. Section 6 presents the performance simulation results. Finally, Section 7 concludes the paper.

## 2. Related works

Recently, the technique of mobile cloud computing has been widely studied. For instance, an extensive survey of mobile cloud computing research and a taxonomy based on some key issues is presented in [11–13]. To address the problem of low resource availability in mobile cloud computing, a multi-hop networking system called “MoNet” is introduced, based on which a distributed content sharing protocol is implemented in [14]. In addition, a mobile device is under a lot of pressure when connecting to a remote server due to its limited energy source. To reduce the communication cost between the user and a remote data center, the authors in [15] propose a three-tier cloud system, in which the resource provision can be extended to the edge of the network. The technology of mobile edge computing can be used to reduce transmission latency [16].

What is more, how to reduce energy consumption has received great attention. In [2], a mobile cloud computing model based on the Cloudlet scheme is proposed to save processing energy. In [17], an efficient dynamic resource provisioning scheduler is presented to minimize the computation and communication energy consumption. In addition, an energy-efficient adaptive resource scheduler based on Networked Fog Centers is proposed in [18], which designs and tests the performance of a distributed and adaptive resource management controller [19]. To improve the performance of mobile applications and reduce the energy consumption, the offloading issue is investigated in [20–21]. In addition, to address scalability and time constraints, the authors in [22] propose an cloud-centric, multi-level authentication as a service approach.

The idea of cooperation and dynamic resource sharing has emerged as an extension for cloud computing [23–24]. In [25], the “Crowd-Cloud” architecture is proposed by integrating the sensing and processing capabilities of the dynamic mobile cloud, which can minimize the overall energy consumption and efficiently utilize the available resources. In [26], a new approach is proposed based on “a local crowd” composed by the vehicles near the event, which has a lower delay of offloading data than from remote cloud. To stimulate more rational participants participate in the “Crowd” and achieve good service quality, an incentive mechanism is designed using a Stackelberg game [27], and data quality is considered in the design of incentive mechanism [28]. Considering the trustworthiness of participants, the authors in [29] present a thorough performance study of vote-based trustworthiness with trusted entities.

Illuminated by the idea of sharing and resource expansion, we consider the resource sharing based on Crowd-funding among various devices in the network. These devices can be seen as the service provider of mobile cloud applications. Furthermore, for the resource cooperation, an appropriate resource management scheme and an impartial revenue sharing method is necessary, which will be discussed in this paper.

## 3. The Framework of Crowd-funding

In this section, we first introduce the application scenario for mobile cloud computing under Crowd-funding model. Then, we introduce the specific problem background of this paper.

### 3.1 Design of Crowd-Funding

**Fig 1:**
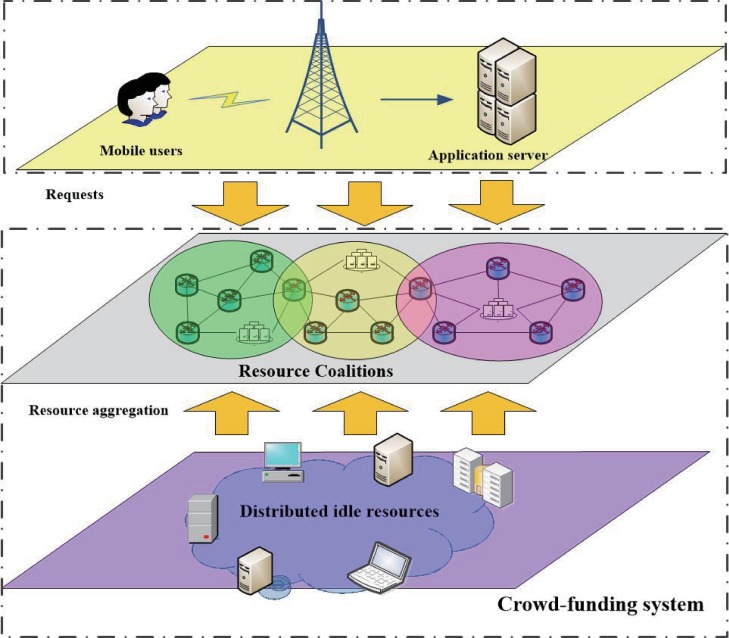
Crowd-funding for mobile cloud computing scenario.

**Fig 1** shows the main application scenario for mobile cloud computing, in which we consider three principal parts: mobile user, application server, and Crowd-funding system. Without a loss of generality, the resources of mobile user are assumed to be limited. When the mobile user needing the support of Crowd-funding system, they will send a request to the application server, and then the application server will contacts the Crowd-funding system to request resource support. To improve computing speed and conserve battery power, most of the work of mobile users will be offloaded to the cloud computing platform (i.e., Crowd-funding system), with only a minor part of the work left for local processing (i.e., the mobile user). In general, Crowd-funding provides an execution environment and appropriate resources for mobile applications.

Crowd-funding is composed of many distributed devices with spare resources, which can provide support for mobile cloud applications. Although the amount of resource in a single device might be limited, it would be considerable if many devices can cooperate with each other and form a resource coalition. For the resource providers are distributed in the network, Crowd-funding is more suitable for applications that need a small quantity of data interaction

### 3.2 Problem statement and parameter description

**Fig 2:**
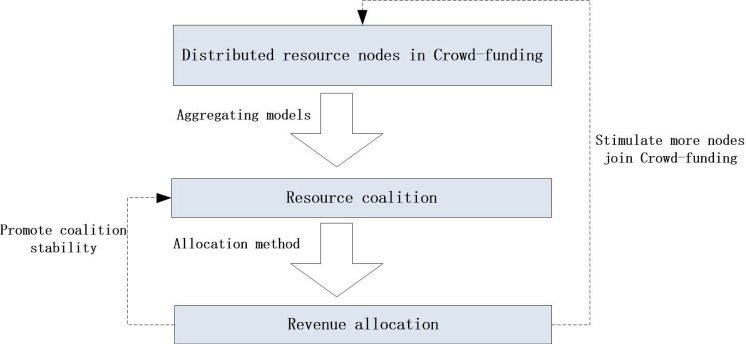
Framework of Crowd-funding.

For simplification, in this paper, the device that can provide resources to support mobile applications are regarded as the "resource node" hereafter. The resource nodes in Crowd-funding system are sporadically distributed in the network, whose resource capacity may be too limited to support some resource-intensive mobile applications alone. Hence, to meet the resource demand of mobile applications, we consider the cooperation among these resource providers by form a logical resource coalition. For various requests, we would like to find out the corresponding suited coalition according to the specific requirement. Therefore, a reasonable resource aggregation model becomes critical. Moreover, rational resource providers should achieve some reasonable benefit for their services, which can make them provide resources continually and then promote the coalition's stability [10]. Thus, to promote the coalition's stability and stimulate more nodes to join the Crowd-funding simultaneously, an impartial revenue allocation method is necessary. The framework of Crowd-funding is shown in **Fig 2**, which presents two questions to be resolved:

- How to aggregate resources and form an appropriate resource coalition according to the specific requirements of applications
- How to fairly allocate revenue among the resource providers in the resource coalition

**Definition 1: Resource coalition**—Resource coalition is defined as "the resource cooperation group composed by some resource nodes, which can be used to perform a specific application or an application combination and be denoted by *C*_*k*_ ".

In this paper, the set of mobile cloud application types is denoted as *D* = {1,2,…,*d*}. The set of resource nodes in Crowd-funding system is represented as *M* = {1,2,…,*m*}. Without a loss of generality, we consider two types of resources: computing resources and storage resources. In addition, the meanings of necessary symbols are shown in **Table 1**. When the request arrives, Crowd-funding systems will allocate a resource coalition to provide service, and achieve some revenues in return.

**Table 1:** Parameters definition.

Symbol	Description
$P_{m}^{CR}$	The amount of computing resource provided by node *m*, *m* ∈ *M*
$P_{m}^{SR}$	The amount of storage resource provided by node *m*, *m* ∈ *M*
$P_{d}^{CR}$	The demand for computing resources of one application instance *d*, *d* ∈ *D*
$P_{d}^{SR}$	The demand for storage resources of one application instance *d*, *d* ∈ *D*
*V*_*d*_	The unit revenue received by executing one application instance *d*, *d* ∈ *D*
*K*_*d*_	The instance number of application *d* in the current request
*C*_*C*_	The unit cost of providing one computing resource
*S*_*C*_	The unit cost of providing one storage resource
*v*(*C*_*k*_)	The revenue achieved by coalition *C*_*k*_
*NA*(*C*_*k*,_, *d*)	The instance number of application *d* that coalition *C*_*k*_ can serve at the same time
*P*^*CR*^(*C*_*k*_)	The computing resources provided by *C*_*k*_
*P*^*SR*^(*C*_*k*_)	The storage resources provided by *C*_*k*_

On the resource providing side, the total revenue should be the revenue sum of all types of applications. For one type of application, the revenue is the product of unit revenue and the instance number of this application. Thus, the revenue of coalition can be calculated as Eq (1). For Eq (2) and Eq (3) $P_{m}^{CR}$, and $P_{m}^{SR}$ are the amount of computing resource and the amount of storage resource in an arbitrary node *m*, respectively. Thus, the total quantity of computing resources and storage resources in *C*_*k*_ is the sum of quantities in every node.

$$v(C_{k})=\sum_{d\in D}NA(C_{k},d)*V_{d}$$ (1)

$$P^{CR}(C_{k})=\sum_{m\in C_{k}}P_{m}^{CR},m\in C_{k}$$ (2)

$$P^{SR}(C_{k})=\sum_{m\in C_{k}}P_{m}^{SR},m\in C_{k}$$ (3)

On the resource request side, each request may include more than one type of application. More importantly, the resource demand for each type of application may be different. For one instance of application *d*, the demand for computing resource and storage resource is expressed by *RCR d* and *RSR d*. If the instance number of application *d* is *K*_*d*_, the demand for computing resource of application *d* is *K*_*d*_ * *RCR d*. Thus, the total demand for computing resource of all applications should be calculated as Eq (4). The demand for storage resource is the same as shown in Eq (5).

$$TCR=\sum_{d\in D}{K_{d}*R}_{d}^{CR}$$ (4)

$$TSR=\sum_{d\in D}{K_{d}*R}_{d}^{SR}$$ (5)

## 4. The Resource Aggregation Models for Crowd-funding

In Crowd-funding system, there are many resource nodes providing resource support for mobile cloud applications. Considering different requests, we need to select different resource nodes and form different resource coalition according to different specific requirement. For requests without any special QoS requirement, we design a resource aggregation model to find out the resource coalition with the highest average revenue of unit resource (i.e., “Max-CR”). For requests with some special requirements for service-level, the coalition's QoS level should be treated as the first-class objective. Thus, the coalition's QoS level-based model (i.e., “Max-CQ”) is proposed.

### 4.1 “Max-CR” Model for resource aggregation

As shown in **Table 1**, *P*^*CR*^(*C*_*k*_) and *P*^*SR*^(*C*_*k*_) represent the amount of computing resources and storage resources in coalition *C*_*k*_, respectively. Note that *TCR* and *TSR* denote the demands for storage and computing resources. In this paper, “Max-CR” is designed to find out the resource coalition whose average revenue of unit resource is the highest. Consequently, the following objective function will determine the target resource coalition that we want:

$$Max\;Resource\;Revenue(C_{k})=\frac{\sum_{d\in D}V_{d}*K_{d}-(C_{C}*P^{CR}(C_{k})+S_{C}*P^{SR}(C_{k}))}{P^{CR}(C_{k})+P^{SR}(C_{k})}$$ (6)

$$s.t.\;P^{CR}(C_{k})\geq TCR$$ (7)

$$P^{SR}(C_{k})\geq TSR$$ (8)

where ∑_*d* ∈*D*_ *V*_d_*K_*d*_ denotes the gross income of coalition *C*_*k*_. Moreover, (*C*_C_*P^*CR*^(*C*_*k*_)+*S*_*C*_ * *P*^*SR*^(*C*_*k*_)) stands for the total cost of coalition *C*_*k*_. Thus, the net revenue of coalition *C*_*k*_ can be represented as the difference value of gross income and total cost. Furthermore, the average revenue of unit resource in coalition *C*_*k*_ is calculated as (6). The constraints in (7) and (8) can ensure that the resources provided by coalition *C*_*k*_ will meet the resource demand.

### 4.2 “Max-CQ” Model for resource aggregation

QoS is regarded as an important performance indicator for measuring the performance of cloud systems; this indicator is extremely important to users. Therefore, the proposed “Max-CQ” model is designed based on the QoS criteria of nodes and is aimed at finding out the resource coalition with the highest QoS level. Before introducing the objective function of “Max-CQ” Model, we need to describe the calculation method of QoS level.

#### 4.2.1 The calculation method of QoS level

In this section, we will introduce QoS criteria of resource nodes firstly. Then we will introduce that how to express and normalize the QoS level of resource coalition.

1 In this paper, the QoS is measured using three types of parameters: Response time, Availability, and Reputation. The three typical QoS criteria are available in **Table 2**. Furthermore, the QoS level of a node *m* is described as <*t*, *a*, *r*>.

**Table 2:** Typical QoS criteria of node.

QoS Criterion of Nodes	Description	Unit
Response time *t*(*m*)	The execution duration between the moment a request arrives and the moment a result returns	ms
Availability *a*(*m*)	The probability that a node is accessible	percent
Reputation *r*(*m*)	The trustworthiness degree of a node	percent

2 For coalition *C*_*k*_, the parameter *m* denotes an arbitrary node in *C*_*k*_. The integrating functions of QoS criteria for coalitions are described in **Table 3** [9]. Furthermore, *t*(*C*_*k*_), *a*(*C*_*k*_) and *r*(*C*_*k*_) indicate the response time, availability and reputation of coalition *C*_*k*_, respectively.

**Table 3:** QoS criteria for coalitions.

QoS criterion	Response time (*t*)	Availability (*a*)	Reputation (*r*)
Integrating function	$t(C_{k})={max}_{m\in C_{k}}\;t(m)$	$a(C_{k})=\prod_{m\in C_{k}}a(m)$	$r(C_{k})={min}_{m\in C_{k}}\;r(m)$

3 Note that the criteria need to be normalized to achieve a uniform measurement. In the following, we employ the Simple Additive Weighting (SAW) technique, which has been widely used for performing multiple dimensions computation [30]. The QoS criteria can be classified into two types: positive criteria and negative criteria.
  - Positive criteria are defined as the increases that will be beneficial to the users, such as in availability and reputation.
  - Negative criteria, on the other hand, are described as the decreases that will benefit the users, such as in response time.

Eq (9) and Eq (10) express the normalization formulations of positive and negative criteria, respectively; *f + x*(*C*_*k*_) and *f—x*(*C*_*k*_) are normalized values of the *x*-th criterion for coalition *C*_*k*_. Furthermore, *q*_*x*_(*C*_*k*_) expresses the original value of the *x*-th QoS criterion for coalition *C*_*k*_. Moreover, *max q*_*x*_ and *min q*_*x*_ denote the maximum value and minimum value of the *x*-th QoS criteria.

$$f_{x}^{+}(C_{k})=\left\{ \begin{array}{l} \frac{q_{x}(C_{k})-min\;q_{x}}{max\;q_{x}-min\;q_{x}},\;maxq_{x}-minq_{x}\neq 0\\ 1\;,\;maxq_{x}-minq_{x}=0 \end{array}\right.$$ (9)

$$f_{x}^{-}(C_{k})=\left\{ \begin{array}{c} \frac{max\;q_{x}-q_{x}(C_{k})}{max\;q_{x}-min\;q_{x}},\;maxq_{x}-minq_{x}\neq 0\\ 1\;,\;maxq_{x}-minq_{x}=0 \end{array}\right.$$ (10)

Moreover, the user preferences for different criteria are considered in this paper. Note that *ρ*_*x*_ reflects the user preference for different QoS criteria. The number of QoS criteria is denoted by *w*; the QoS level of *C*_*k*_ is calculated by solving:

$$QoS\;(C_{k})=\sum_{x=1}^{w}\rho_{x}f_{x}(C_{k}),\;\sum_{x=1}^{w}\rho_{x}=1$$ (11)

#### 4.2.2 Objective function of “Max-CQ” model

We consider three types of QoS criteria in this paper; thus, *w* is set to 3. The objective of the “Max-CQ” model is to find out the resource coalition with the highest QoS level. In this case, the objective function will determine the target resource coalition that we want:

$$\begin{array}{c} Max\;QoS\;(C_{k})=\sum_{x=1}^{3}\rho_{x}f_{x}(C_{k})=\rho_{1}f_{1}^{-}[t(C_{k})]+\rho_{2}f_{2}^{+}[a(C_{k})]+\rho_{3}f_{3}^{+}[r(C_{k})]\\ \;{=\rho }_{1}f_{1}^{-}[{max}_{m\in C_{k}}\;t(m)]+{\rho_{2}f}_{2}^{+}[\prod_{m\in C_{k}}a(m)]+\rho_{3}f_{3}^{+}[{min}_{m\in C_{k}}\;r(m)] \end{array}$$ (12)

$$s.t.\;P^{CR}(C_{k})\geq TCR$$ (7)

$$P^{SR}(C_{k})\geq TSR$$ (8)

$$t(n_{i})\leq T,\;{\forall \;n}_{i}\in C_{k}$$ (13)

$$a(n_{i})\geq A,\;{\forall \;n}_{i}\in C_{k}$$ (14)

$$r(n_{i})\geq R,\;{\forall \;n}_{i}\in C_{k}$$ (15)

where *ρ*_1_, *ρ*_2_, *ρ*_3_ represent the user preference for response time, availability and reputation, respectively. Without a loss of generality, the response time is assumed to be a negative criterion, while the availability and reputation are two positive criteria. The constraints in (7) and (8) ensure that the resource quantity in coalition *C*_*k*_ can meet the resource demand. Furthermore, the constraints in (13)-(15) are to ensure that every node in *C*_*k*_ can satisfy the constraints of a user's request.

### 4.3 Aggregation Algorithm

**Fig 3:**
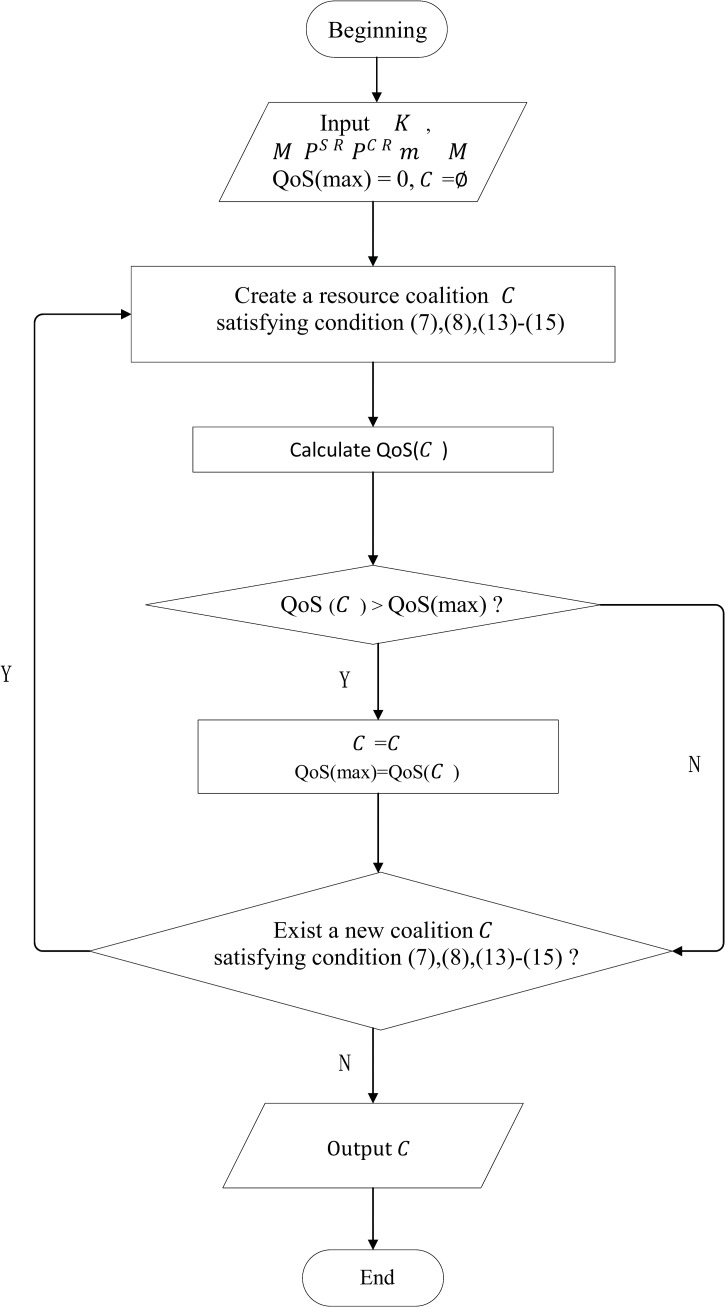
The algorithm flow for Max-CQ model.

To realize the objectives of “Max-CR” model and “Max-CQ” model, that is to find out the target coalition, a specific implementation algorithm is presented in this section. For “Max-CR”, the objective is to seek out the resource coalition who can achieve the maximum average revenue of unit resource. For “Max-CQ”, the objective is to seek out the resource coalition who can provide the best service level. The target resource coalition is expressed as *C*_*g*_. Although the aggregation objectives of two models are different, the algorithm flow is similar. Take Max-CQ for example, the algorithm flow is shown in Fig 3.

## 5. The Revenue allocation method

In return, the revenues of coalition *C*_*g*_ generated by supporting application instances should be shared among the resource providers. To promote the resource providers to continually support the mobile applications and stimulate more nodes to participate in the Crowd-funding system, it is necessary to present a reasonable revenue allocation method. Note that the resource contribution of each node is different, thus revealing a key problem: how to allocate the revenues among these nodes impartially.

In this paper, all the nodes in the resource coalition are selfish, seeking to maximize their own benefit. The relationship among these nodes can be regarded as a cooperative game. The coalition accords with the super additivity, as shown in **definition 2**. Note that every sub-coalition of *C*_*g*_ cannot provide more resources or make more revenue than *C*_*g*_. Thus, we can obtain some ideas from the solution of the Shapley value in a cooperative game for allocating the revenue among nodes.

**Definition 2: Super additivity—**In a cooperative game < *N*, *v* >, for every sub-coalition *S* and *T* (*S* ∩ *T* = *Φ*), if they have a relationship in the form of *v*(*S*) + *v*(*T*)≤ *v* (S ∪ T), we would regard the cooperative game < *N*, *v* > as super additive.

**Definition 3: Shapley Value**—The Shapley value is a concept of game theory that is used to allocate benefits among the members of a mutual cooperation. In this paper, we introduce the Shapley value to provide a fair revenue management scheme for the resource nodes in coalition *C*_*g*_. The Shapley value of any node *m*, *m* ∈ *C*_*g*_, can be denoted by *ψ*_*m*_(*v*) as [31]:

$$\psi_{m}(v)=\sum_{S\subseteq C_{g}}\frac{(|s|-1)!(|C_{k}|-|S|)!}{|C_{k}|!}[v(S)-v(S/\{m\})]$$ (16)

where *ψ*_*m*_(*v*) denotes the revenue for node *m* and *S* is the sub-coalitions of *C*_*g*_. The characteristic function *v* (*) represents the revenue of coalition *, calculated by solving Eq (1), and | * | is the number of nodes in coalition *.

To illustrate the Shapley value more clearly, one example is provided as follows: we assume that *C*_*g*_ comprises three nodes, i.e., *C*_*g*_ = {*node*1, *node*2, *node*3}. Thus, *S* represents six sub-coalitions: {*node*1}, {*node*2}, {*node*3},{*node*1, *node*2}, {*node*2, *node*3}, and {*node*1, *node*3}, with |*S*| being, respectively, 1, 1, 1, 2, 2, and 2. Furthermore, | *C*_*g*_ | is 3. For example, the revenue of *node*1 can be calculated as follows:

$$\begin{array}{c} \Phi_{1}(v)=\frac{(1-1)!(3-1)!}{3!}[v\{1\}-v(\emptyset )]+\frac{(2-1)!(3-2)!}{3!}[v\{1,2\}-v\{2\}]\\ \;+\frac{(2-1)!(3-2)!}{3!}[v\{1,3\}-v\{3\}]+\frac{(3-1)!(3-3)!}{3!}[v\{1,2,3\}-v\{2,3\}] \end{array}$$ (17)

## 6. Performance evaluation

### 6.1 Simulation scenarios

For the sake of simplification, the costs of one storage resource and one computing resource are set as 1 monetary unit (MUs). As shown in **Table 4**, there exist two application types: speech recognition and image retouching applications [32]. The speech recognition application (Application 1) requires 22 computing resources and 12 storage resources, and the revenue of running this application is 500 monetary units (MUs) per instance. The image retouching application (Application 2) requires 10 computing resources and 50 storage resources, and the revenue of running this application is 800 monetary units (MUs) per instance. Some experiments are based on some combinations of Application 1 and Application 2, as shown in **Table 5**. Finally, for the resource provider, the amounts of computing resources and storage resources in each node set have a Gaussian distribution and a mean value within the range [2, 50] and [8, 75]. The ranges of response time, availability and reputation are [0.1, 5], [0.74, 0.99], and [0.55, 0.91], respectively.

**Table 4:** Information of applications.

Application	computing resource	storage resource	Revenue
Speech recognition application (Application 1)	22	12	500 MUs
Image retouching application (Application 2)	10	50	800 MUs

**Table 5:** Application combinations.

Case No.	1	2	3	4	5	6	7	8	9	10	11	12
Number of Application 1	1	1	1	1	1	1	2	3	4	5	6	7
Number of Application 2	1	2	3	4	5	6	1	1	1	1	1	1

### 6.2 Numerical Results

1. In the proposed models for Crowd-funding, we employ “average revenue of unit resource” as the performance indicator to evaluate a models' impact on resource revenue. The meaning of “average revenue of unit resource” can be explained as the average net income of unit resource in the coalition. In the following, “average revenue of unit resource” is defined as the ratio of the coalitions' net revenue to the amount of total resources, as shown in Eq (6). Furthermore, “resource utilization rate” can indicate the utilization efficiency of resources, which can be described as the ratio of consumed resources to the coalition's total resources. Note that “SP” is used as a comparison model, proposed in [16], designed for allocating resources under the background of resource cooperation in mobile cloud computing. To establish a similar simulation scenario with [33], in this paper, we assume three of the resource providers as the fixed cooperators and other resource providers as the resource expansion parts of the cooperators. For the “SP” model, the penalty cost of per unsupported speech recognition application and per unsupported image retouching application is 300MUs and 500MUs, respectively [33].

**Fig 4:**
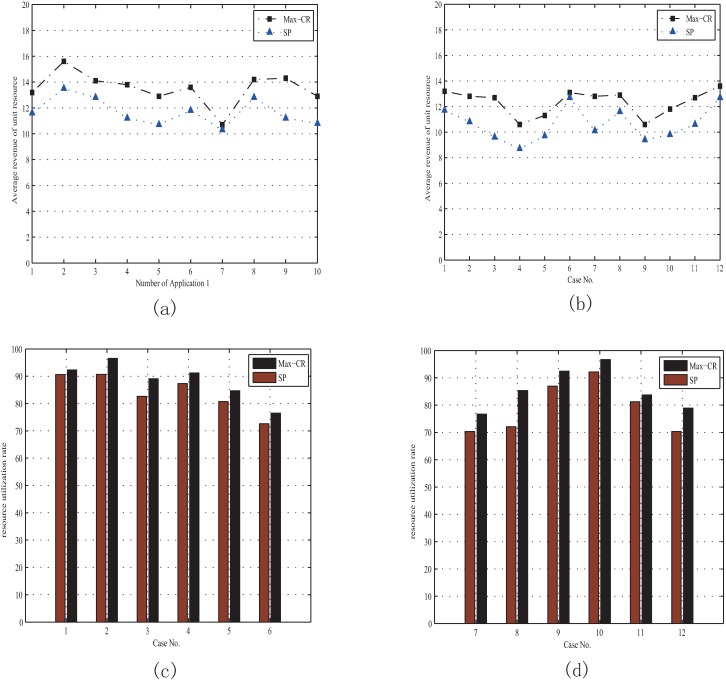
Performance evaluation for “Max-CR” with sufficient resource provision. (a) Average revenues of unit resource with different numbers of Application 1. (b) Average revenues of unit resource with different Case No. (c) Resource utilization rates when the Case No. from 1 to 6. (d) Resource utilization rates when the Case No. from 7 to12.

Fig 4 shows the performance of “Max-CR” with variant application combinations when there are enough resources nodes in the system. Fig 4(A) and Fig 4(B) illustrate that the average revenue of unit resource with “Max-CR” can often exceed 12 MUs regardless of how many applications requesting simultaneously. The average revenue of unit resource with “Max-CR” is always higher than that with “SP”. For “Max-CR”, it always can search out the resource coalition with the maximum average revenue of unit resource as long as there are enough different resource nodes to choose from. Furthermore, higher average revenues can make the coalition more stable and stimulate more nodes to join the Crowd-funding. In addition, for the resource demand and gross income are invariant when the number of requested resources is the same, so the coalition's net revenue depends on the resource costs as shown in Eq (6). To make the resource coalition achieve more net revenue, we should decrease the coalition's cost, which means decreasing the redundant resources. “Max-CR” can determine a node combination carrying the fewest redundant resources, but “SP” cannot. Thus, the ratio of consumed resources to total resources (expressed as "resource utilization rates") with “Max-CR” is always higher than that with “SP” as shown in Fig 4(C) and Fig 4(D).

**Fig 5:**
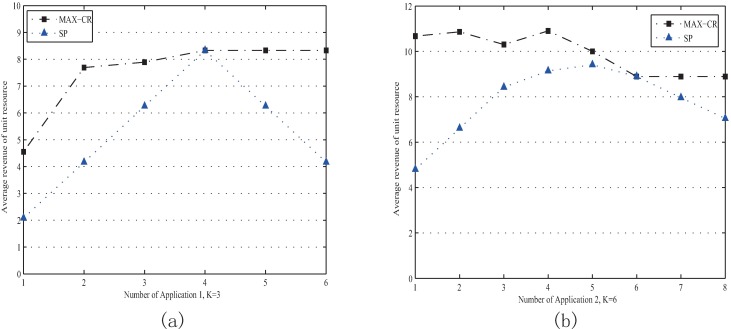
Performance evaluation for “Max-CR” with insufficient resource provision. (a) Average revenues of unit resource with different numbers of Application 1 when K = 3. (b) Average revenues of unit resource with different numbers of Application 2 when K = 6.

In this section, *K* represents the number of total resource nodes in Crowd-funding system. When the resource provision is enough, that is when the number of applications is less than 4 as shown in Fig 5(A), the average revenue of unit resource of “Max-CR” are higher than those of “SP”. When the number of applications is exceeding 4, the resource provision of Crowd-funding system is insufficient and only part of the application can be supported. Thus, the average revenue of unit resource will be changeless at 8.3 MUs no matter how many applications request. The average revenues of resource with “SP” are less than those with “Max-CR” after the number of applications exceeding 4, because there is a penalty mechanism in “SP” model when there are applications cannot be supported. Similarly, when *K* is 6, the resource provision is insufficient when the number of applications is exceeding 6, and the average revenue of unit resource with “Max-CR” will be maintained at about 8.8 MUs, as shown in Fig 5(B).

**Fig 6:**
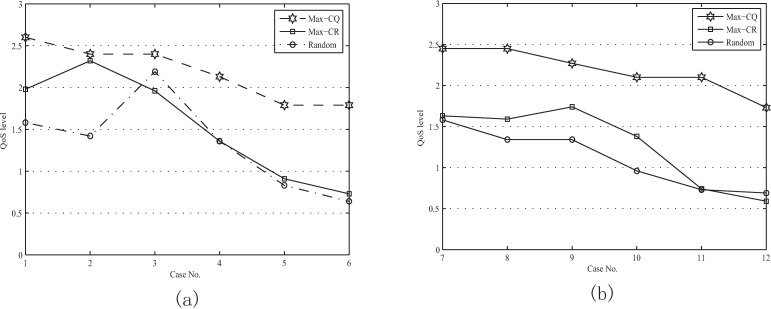
Performance evaluation for “Max-CQ”. (a) QoS levels when the Case No. from 1 to 6. (b) QoS levels when the Case No. from 7 to 12.

2. In the following, the “QoS level” is used to evaluate the coalition's service level, as defined by Eq (11). “Max-CQ” is a resource aggregation model, which is designed to search out the resource coalition with the highest QoS level. “Random” is also a resource aggregating method, which forms a resource coalition at random in the case of meeting the resource demand of applications. The QoS level of a coalition depends on the QoS level of nodes that make up it. “Max-CQ” can search out the nodes with high QoS levels to form the resource coalition, thus the coalition's QoS levels formed by “Max-CQ” model are always higher than those formed by “Max-CR” and “Random”, as shown in Fig 6(A) and Fig 6(B). However, more and more nodes need to join the coalition to meet the resource demand if there are more and more application requests, which may result in the decrease of the coalition's response time, availability and reputation. Thus, we can find that the coalition's QoS level decreases from about 2.5 to less than 2 with the number of applications increasing.

3. In this section, we set up a new experimental scenario based on Hadoop platform, which consists of 12 nodes. One of the nodes acts as the Crowd-funding broker (i.e., management node) for running two aggregation methods, and the other nodes are used to perform the jobs. We consider some different combinations of real “WordCount” job and “PageRank” job as the application request, which is shown in Table 6. The experiments are based on different scales of input data, which required different completion time. Furthermore, we use Ganglia system to monitor the status of this cluster to obtain the experiment results.

**Table 6:** Application combinations.

Case No.	Number of PageRank	Number of WordCount	Scale of input data	Expected completion time
**1**	10	10	200M	85s
**2**	10	30	400M	100s
**3**	20	40	600M	110s
**4**	30	50	800M	130s
**5**	40	60	1000M	160s

**Fig 7:**
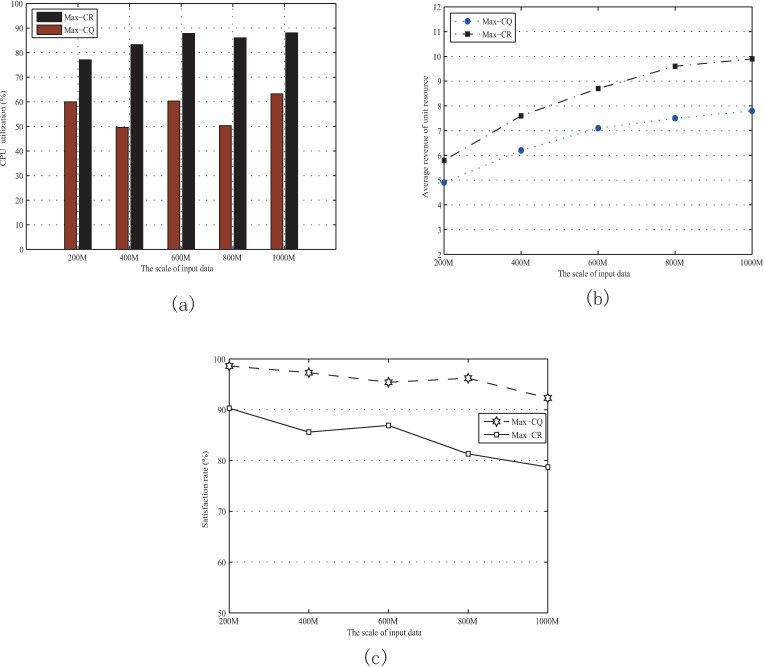
Performance evaluation under Hadoop platform. (a) CPU utilizations with different scales of input data. (b) Average revenues of unit resource with different scales of input data. (c) Satisfaction rates with different scales of input data.

From the data monitored by Ganglia, we can find that the CPU utilization with Max-CR is about twenty four percent higher than that with Max-CQ as shown in Fig 7(A). In addition, the average revenue of unit resource with Max-CR is always higher than that with Max-CQ. For Max-CR, the average revenue of unit resource is increasing from 5.85 MUs to 8.9 MUs as the expansion of input data, and gap of average revenue between Max-CQ and Max-CR is increasing as shown in Fig 7(B). Furthermore, “Satisfaction rate” can be defined as the probability that jobs can be completed within the expected completion time. According to our statistics, the Satisfaction rate with Max-CQ is about 10%-17% higher than that with Max-CR, as shown in Fig 7(C).

## 7. Conclusion

With the rapid development of mobile cloud computing techniques, a flexible resource-management scheme is necessary to meet the different requirements of a growing number of mobile cloud applications. In this paper, we proposed a new scheme called "Crowd-funding", in which devices with spare resources in a network can cooperate with each other to share their resources. Meanwhile, these resource providers can achieve some revenues as benefits in Crowd-funding. It is necessary to realize an effective scheduling between the cloud applications and the resource nodes in the resource coalition; this will be our future work.

## Supporting Information

S1 File Algorithm flow for Max-CR model. (PDF)
