# Supplementary material for: Crowd-Funding: A New Resource Cooperation Mode for Mobile Cloud Computing
Source: PLoS One. 2016 Dec 28;11(12):e0167657. doi: 10.1371/journal.pone.0167657 (PMC5193344; doi:10.1371/journal.pone.0167657)

Algorithm flow for Max-CR model

This figure is the supplementary information of Section 4.3. In order to simplify the content in the main body, we only introduce an algorithm flow for Max-CQ resource aggregation model. Therefore, we supplement the algorithm flow for Max-CR model in this file.

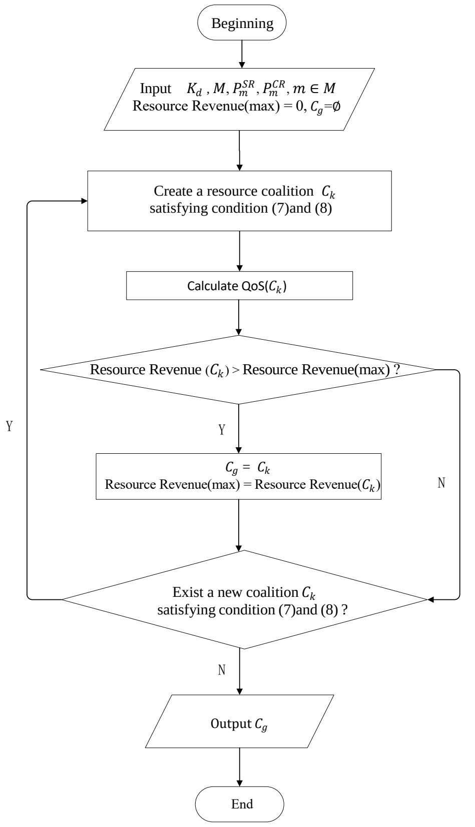

Supplement: S1 File — (PDF) [file pone.0167657.s001.pdf]
